# Comparison of patient experiences of the osteoarthritis consultation with GP attitudes and beliefs to OA: a narrative review

**DOI:** 10.1186/1471-2296-15-46

**Published:** 2014-03-19

**Authors:** Zoe Paskins, Tom Sanders, Andrew B Hassell

**Affiliations:** 1 Arthritis Research UK Primary Care Centre, Keele University, Keele ST5 5BG, UK; 2 School of Medicine, Keele University, Keele ST5 5BG, UK

**Keywords:** Osteoarthritis, Primary health care, General practitioners, Communication

## Abstract

**Background:**

Osteoarthritis (OA) is a common cause of disability and consultation with a GP. However, little is known about what currently happens when patients with OA consult their GP. This review aims to compare existing literature reporting patient experiences of consultations in which OA is discussed with GP attitudes and beliefs regarding OA, in order to identify any consultation events that may be targeted for intervention.

**Methods:**

After a systematic literature search, a narrative review has been conducted of literature detailing patient experiences of consulting with OA in primary care and GP attitudes to, and beliefs about, OA. Emergent themes were identified from the extracted findings and GP and patient perspectives compared within each theme.

**Results:**

Twenty two relevant papers were identified. Four themes emerged: diagnosis; explanations; management of the condition; and the doctor-patient relationship. Delay in diagnosis is frequently reported as well as avoidance of the term osteoarthritis in favour of ‘wear and tear’. Both patients and doctors report negative talk in the consultation, including that OA is to be expected, has an inevitable decline and there is little that can be done about it. Pain management appears to be a priority for patients, although a number of barriers to effective management have been identified. Communication within the doctor patient consultation also appears key, with patients reporting a lack of feeling their symptoms were legitimised.

**Conclusions:**

The nature of negative talk and discussions around management within the consultation have emerged as areas for future research. The findings are limited by generic limitations of interview research; to further understanding of the OA consultation alternative methodology such as direct observation may be necessary.

## Background

Osteoarthritis (OA) is the most common arthritis, a common cause of disability and frequent cause of consultation with a GP. OA is predominantly managed in primary care, and comprehensive guidance suggests that much can be done to alleviate symptoms from osteoarthritis; a combination of therapeutic options including pharmacological and non-pharmacological treatments are recommended [1-5].

However, evidence suggests that for many patients with OA, effective interventions appear not to have been adopted.

Firstly, various studies that have evaluated OA treatment according to guidance generally report low uptake of ‘core’, first line non-pharmacological measures, such as weight loss advice, provision of patient information and aerobic and strengthening exercise [6-9]. The reasons for this are not clear, although likely to be influenced by a number of variables including awareness of the guidance [10].

Secondly, patient reported measures suggest the condition may be inadequately treated. A large survey of patients commissioned by Arthritis Care in the UK reports that patients experience long delays before they are diagnosed and that they don’t feel OA is a priority for the healthcare system [11]. More worryingly, the survey suggests nearly three quarters of people with OA are living with constant pain, with 40% of those surveyed reporting their treatment either ‘not very’ or ‘not’ effective.

One significant barrier to treatment in OA, is choosing not to seek healthcare. A recent review suggests a number of patients with painful OA chose not to visit their GP; reasons for this include believing that OA is a normal part of ageing or perceiving a negative response from the GP [12]. However, when patients do visit their GP, little is known about what actually happens in the consultation. Understanding more about the consultation may further explain the reasons for the apparent gap between the guidance, and the positive view conveyed by OA experts and the apparent ‘real world’ experience.

This review aims to illuminate this issue, by summarising and reviewing existing literature regarding patients’ experiences of consultations regarding osteoarthritis in primary care, and comparing this with literature reporting GP attitudes and beliefs to OA. Understanding patient experience, while considering GPs views, may provide insight into aspects of the consultation that could be targeted for intervention with the aim of enhancing OA care, and this review is conducted in the context of a larger programme of work with that aim.

## Methods

An initial literature search, performed as a scoping exercise identified relevant research using a range of methods including interviews, focus groups and surveys. Due to the diversity of studies, a narrative review was therefore felt to be most appropriate to confer the flexibility needed to review the relevant literature. A narrative review is described as a ‘first generation ‘traditional’ literature review; narrative reviews have a useful place for identifying themes and gaps in the literature and for informing direction of further research [13]. This review is underpinned by a systematic literature search; combining narrative and systematic methods has value in enhancing transparency and rigour of narrative reviews. The literature search was undertaken by searching relevant databases (Medline, CINAHL, Psychinfo, EMBASE and Google scholar), reference checking, manual searching of relevant journals and recommendations from experts. The search terms used specified the population of interest (patients with osteoarthritis), the setting (the primary care consultation with a general practitioner) and ‘experiences’. Search terms used are shown in Table 1. All MeSH headings relating to OA were used with the exception of OA spine; this review aimed to summarise the experiences of those with peripheral joint OA, and not back pain.

**Table 1:** Search terms used to identify experience of GPs and patients consulting with OA

**1 Setting: consultation in primary care**	**2 Population: patients with OA**	**3 Experience**
**Primary health care**	**Osteoarthritis**	**Qualitative research**
**Family practice**	**Osteoarthritis, knee**	Interview
**General practice**	**Osteoarthritis, hip**	Observation
**Family physicians**	**Arthritis**	Theme*
**General practitioners**		Finding
Consult*		Experience*
		View*
		Attitude*
		Belief*

Nb. Terms in bold are MesH headings. *Used as a ‘wildcard’ to represent any character.

The initial research question aimed to compare patient and doctor consultation experiences; however, the first literature search, performed as a scoping exercise, revealed that papers exploring GPs’ perspectives addressed more abstract components of ‘experience’, and tended to report attitudes and beliefs, rather than ‘experience’ of consultations, per se. For this reason, attitudes and beliefs were added to the search string, and the research question changed accordingly. As in qualitative research generally, this review seeks to describe a range of phenomena, and with this in mind, inclusion criteria were deliberately non-restrictive. Papers were included if any of the empirical data in the results related to patient consultation experience, or GPs’ attitudes and beliefs regarding OA. However, only the findings relating to consultation experience or GP attitudes and beliefs were extracted for inclusion in the review. Quantitative studies reporting GP consultation behaviours only were excluded, for example, medical record reviews, unless additional methodology elicited attitudes and beliefs e.g. free text responses on a survey or additional GP interviews.

Papers were included if they concerned patients with a diagnosis of OA or if the population studied were aged over 45 and had a clinical syndrome of chronic peripheral joint pain without a specific clinical diagnosis of OA. These were included with the assumption that the majority of those included were likely to represent people with OA; in primary care research a clinical rather than radiographic indicator or diagnosis may be more pragmatic, and there is high discordance in the use of the label osteoarthritis [14]. The only exclusion criteria were as follows: non-English language paper; consultation experiences not described (patients); attitudes or beliefs not described (GPs).

To appraise the evidence, no single tool was appropriate for the range of methodologies; however, qualitative research appraisal was informed by the CASP tool [15]. Key themes were extracted from the relevant findings of the included papers by the first author and a narrative review approach [13] applied to the results.

## Results and discussion

**Figure 1:**
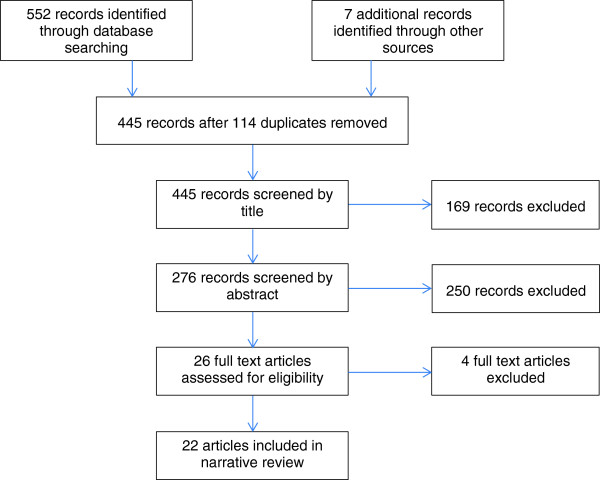
PRISMA flowchart detailing paper selection.

The search identified 552 papers, of which 22 papers were identified as relevant to the review. A PRISMA flow diagram demonstrating selection of papers in detailed in Figure 1. One of the four papers excluded at full text stage was a conference abstract that repeated findings of a paper already included; the other three did not describe consultation experience. The majority of included papers represented UK research (13) with the remainder constituting North American (5), European (3), and Australian (1) studies. The majority of studies evaluated patient experience (12), with the remainder investigating GP views (5) or a combination of the two (5). The majority of included studies used predominately qualitative methodology (interviews: 15; focus group: 5). A summary of the papers identified is shown in Table 2 including a summary of each study aim, the methods used, the relevant findings and limitations.

**Table 2:** Summary of papers identified exploring consultation experiences in OA

**First author, year, country**	**Participants**^**1**^	**Methods**^**2**^	**Aim**	**Extracted findings relating to consultation experience and/or GPs attitudes and beliefs**	**Comments and limitations**
**Alami (2011),**France [24]	81 patients, 11 GPs, 6 Rheum, 4 Orth, 4 Alt Med	Interviews	To explore views on management and barriers to improvement	Patients report importance of doctor patient relationship and various barriers to treatment including side effects, fear of addiction, fear of masking pain, and a wish to focus on preventative options. GP’s report range of attitudes including the belief that OA is not a disease. Some patients and GPs identified OA as an area of uncertainty for GPs.	Not always clear which results (health care practitioners) pertained to GPs. No findings in results to support author claims in abstract and conclusion that patients feel they are not taken seriously and that GPs act as ‘technicians’; findings do not entirely match authors’ conclusions.
**Busby (1997),** UK [18]	80 patients, 3 GPs, 1 Rheum	Interviews fieldwork	To understand perceptions and experiences of OA	Patients describe multiple attempts at seeking healthcare, explanations couched in terms of ageing meant OA was inevitable and that nothing could be done. GPs report lack of therapeutic options threatening doctor-patient relationship.	Results in book chapter. Authors’ report findings from GPs don’t constitute ‘a systematic study’. 80 patients but only 7 cited in findings. No reported analysis methods.
**Coar (2004),** UK [23]	9 GPs, 3 Physio, 3 Rheum	Interviews	To explore GP’s beliefs and attitudes regarding OA	Diagnosis and use of ‘wear and tear’ emergent themes. Use of ‘wear and tear’ perceived as acceptable and useful given lack of alternative terms. Evidence of practitioners playing down severity.	MPhil thesis. Author (GP) reports on limitations and influence of interviewing their peers.
**Davis (2004),** USA [34]	57 Patients	Focus groups	To explore barriers to chronic pain management in arthritis	In the theme ‘relationship with healthcare providers’, patients describe unwelcome focus on prescriptions, and miscommunication in the consultation.	Small part of results relevant to this review; ‘Relationship with healthcare providers’ was one of nine emergent barriers to pain management
**De Bock (1992),** Netherlands [35]	14 GPs	Interviews [Medical record review]	To explore GP’s ‘policy’ in managing OA	Marked variance in the perceived importance and management of OA. Authors conclude consensus needed	Small part of results relevant to this review; small focus on interview findings in results. Little information on analysis of qualitative data.
**Gignac (2006),** Canada [17]	53 patients	Focus groups	To compare health experiences of middle aged and older adults with OA	Patients reported being told OA was normal for age, going to get worse, and were encouraged to accept their symptoms. Conversely, patients felt they had more control over the trajectory of OA. Delays in diagnosis reported and insufficient communication around prescriptions	Study design included ‘control’ focus groups which did not appear to add to conclusions or findings.
**Glauser (2011),** USA [33]	152 GPs, 99 NP & PAs	[Vignettes] survey	To examine the knowledge, attitudes and beliefs and practice of GPs regarding management of OA	Most common educational need identified in free text part of survey was around treatment	Small part of results relevant to this review; methods state researchers elicited barriers to care and confidence in managing OA, but only vignette results and educational needs reported in results. As a result, results mainly address ‘practice’ aspect of study aim.
**Grime (2010),** UK [22]	27 patients	Interviews	To explore perceptions of wellness in elderly people with OA	Reports both discordance and acceptance of ‘wear and tear’ used in diagnosis	Small part of results relevant to this review; most of the results relate to everyday activities and not consulting with a doctor.
**Hill (2010),** UK [29]	29 patients	Focus groups	To explore perceptions and experience of treatment and management of hand OA	Patients described dissatisfaction with amount of information, feeling that ‘nothing can be done’, and held perceptions that GPs lacked understanding of the impact of hand OA. Authors conclude some of the findings imply lack of knowledge of treatment options.	Sample included 14 patients from secondary care, and not always clear which setting consultation experiences related to.
**Jinks (2007),** UK [20]	22 patients	Survey interviews	To investigate population and individual needs assessment	Patients report being told their pain is ‘wear and tear’, related to age, to ‘live with it’ and that nothing can be done. Patients also held the view nothing could be done.	Small part of results relevant to this review; most of the results relate to living with knee pain. Patients were > 50 years and had self-reported knee pain, and may not all have had OA.
**Kee (1998),** USA [37]	20 patients	Interviews	To gain an ‘insider view’ of living with OA	The theme ‘staying in charge’ describes patients’ lack of adherence with GP recommended interventions, with examples of miscommunication.	Small part of results relevant to this review; most of the results relate to living with OA.
**Kingsbury (2012),** UK [31]	232 GPs	Survey	To identify GP reported management of OA	GPs described barriers to effective OA management including inability to manage pain adequately, time in the consultation and enabling patients to make lifestyle changes.	Small part of results relevant to this review; most of the findings relate to self-reported GP behaviours. Low response rate
**Lambert (2000),** USA [28]	12 patients, 14 Doctors (including GPs, rheum and others)	Focus groups	To understand views and experience of OA care and expressed needs	Patients value ‘low-tech’ treatments with doctors tending to value medicines and surgery. Doctors report OA as being related to ageing, which patients report as difficult to accept. Doctors reported lack of musculoskeletal training as an issue, and specific educational needs were identified.	Authors do not specify number of GPs, and sample includes other secondary care doctors; not clear which findings relate to GPs.
**Mann (2011),** UK [16]	16 patients, 2 GPs, 1 Rheum, 1 OT, 2 Physio, 4 NPs	Focus groups and interviews	To explore views on provision of care and possible improvements	Patients reported delays in diagnosis, a feeling that ‘nothing was done’, and difficulty knowing when to return to the doctor. Patients reported OA was not a priority and health professionals reported lack of time as an issue. A GP participant reported not perceiving a need for patient information, although the HP as a whole identified a need for more information	Only 2 GP participants.
**McHugh (2007),** UK [27]	21 Patients	Semi-structured interviews	To investigate the experiences of patients on the waiting list for joint replacement	Patients reported hiding their symptoms from their GP after previous negative experiences.	Small part of results relevant to this review; much of the results about living with OA and self-management etc.
**Pitt (2008),** Australia [36]	13 GPs	Focus groups semi-structured interviews	To explore enablers and barriers to referring patients with OA to self-management programmes	A range of referral patterns and attitudes to self-management in OA were uncovered. Barriers to referral included GPs holding the belief that OA was different to other chronic diseases and time in the consultation	Small part of results relevant to this review; attitudes to OA not primary objective of researchers, and so attitudes elicited were only those of relevance to self-management programme referral. Small sample.
**Rosemann (2006),** Germany [32]	20 patients 20 GPs, 20 NPs	Interviews	To identify health care needs and obstacles for improvements	Patients reported pain and fear of disability as their most important concerns that were inadequately addressed in the consultation, with insufficient information about prognosis. Doctors reported resource issues as barrier to effective treatment, while patients reported communication deficits.	Issues of transferability due to healthcare funding in Germany which reportedly does not ‘value’ conservative treatments equally with non-conservative, and due to large number of non-surgical orthopaedic specialists working in primary care. More findings reported from GPs than patients.
**Sanders (2002),** UK [19]	27 patients	Interviews	To examine the meanings of symptoms of OA	Delays in diagnosis reported. Older participants reported down-playing symptoms.	Small part of results relevant to this review; paper concerns general experience of living with OA.
**Sanders (2004),** UK [25]	27 patients	Interviews	To explore barriers to joint replacement	Participants describe being told nothing can be done; often those who asked about surgery reported being told they were unsuitable for various reasons, including age, by their GP	Small part of results relevant to this review; data extracted from one of 3 themes relating to experiences of primary care.
**Thomas (2013),** UK [30]	11 patients	Semi structured interviews	To describe patient experience of seeing their GP with foot OA	Patients described being given little information, felt foot OA was low priority, and felt there was an ‘unwelcome focus on drugs’.	Conference Proceeding, and therefore limited information on findings
**Turner (2007),** UK [26]	31 patients	Interviews	To investigate beliefs about causes of OA	‘Overwhelming majority’ reported no negative psychological reaction to diagnosis. Some patients reported that GPs had reinforced the belief that OA would deteriorate over time.	Small part of results relevant to this review, around the theme of diagnosis.
**Victor (2004),** UK [21]	170 patients	Interviews [Patient diaries, Group sessions]	To explore patients’ perspective on meaning and significance of OA	Participants reported a lack of information that had been given by GPs previously and uncertainty about the nature, self-management and outcomes of OA.	Small part of results relevant to this review; research conducted in the context of a randomised controlled trial therefore only data relating to participants' previous interaction with healthcare was extracted.

^1^Alt Med: GP specialising in alternative medicine; HP: Health professional; NP: Nurse Practitioner or practice nurse; Orth: Orthopaedic Surgeon; PA: Physician assistant; OT: Occupational Therapist; Physio: Physiotherapist; Rheum: Rheumatologist.

^2^Methods in square brackets yielded data that was not extracted for the purposes of this review.

The evidence is grouped below under four themes derived from the included studies: diagnosis; explanations; management of the condition; and the doctor-patient relationship. Patient and doctor perspectives are discussed under each theme.

### Diagnosis

The issues identified around diagnosis predominantly relate to delays in diagnosis and the diagnostic term or phrase used at the time of diagnosis. Patients describe long delays before being diagnosed in both UK and Canadian research [16-18] in addition to difficulty obtaining a diagnosis and ‘relief’ at symptoms being legitimised [19]. There is some evidence to suggest multiple visits prior to receiving a diagnosis may be a particular issue in younger patients [17].

‘Wear and tear’ has been reported by patients as conveying a range of negative meanings including ‘it’s your age’ and ‘nothing can be done for you’ [20], or that the physician who used the term is ‘giving up’ [21]. Busby [18] argues that the connection with ageing results in the phrase conferring inevitability. However, the phrase is not exclusively associated with negative connotations. Grime et al. found participants used it as ‘shorthand for normal bodily change’ and adopt a ‘use it or lose it’ philosophy to exercise; Grime et al. report the latter finding is in contrast to other reported research suggesting patients may avoid activity due to connotations of wear and tear [22].

In one UK study of GPs’ perceptions of OA, GPs reported withholding or ‘playing down’ the diagnosis, using ‘wear and tear’ in preference to osteoarthritis or degenerative arthritis, in order to either avoid upsetting the patient or prevent the adoption of a ‘sick role’ and increased disability [23]. ‘Wear and tear’ was reported by GP participants as a term that may facilitate acceptance on the part of the patient and that saves time; introducing the term osteoarthritis was felt to necessitate a more detailed explanation [23]. In one French study, GPs described their diagnostic priority as identifying inflammatory joint pain, with the precise nature of mechanical pain being considered unimportant and unrelated to treatment [24].

### Explanations and patient information

There are a number of studies in which patients report that they have been told their joint pain/ arthritis is normal for their age [17,18,20,24,25], and is likely to deteriorate over time [17,26]. Similarly, reports of being told ‘nothing can be done’ are common [17-20], and this has been described as a ‘fatalist’ viewpoint. Patients describe being encouraged to accept their symptoms and ‘live with it’ [20].

Some patient narratives do indicate a degree of acceptance of their symptoms and perseverance with daily activities. Beliefs about symptoms being ‘normal for age’ are moderated by shared experiences of friends and family, and the societal view of ageing [19,26]. It is also worthy of note that patients holding beliefs that nothing could be done or that symptoms were ‘just’ age related have reported withholding symptoms from the GP [19,27].

However, there is evidence of patients rejecting the notion that OA is age-related [28], particularly younger adults [17] who may search for alternative explanations [19].

In an interview study with 81 patients with knee OA, a general dissatisfaction with the ‘vague’ information about the condition is reported [24]. Dissatisfaction with the amount of explanation is also reported in other UK studies [29,30], with a feeling that OA is low priority [30]. The lack of precision in explanations has been interpreted as both lack of interest and lack of knowledge on behalf of the doctor [24,29]. Patients reported that more information regarding disease progression may facilitate self-management and coping [16].

Education regarding prognosis has been identified as a particular area of unmet need in patients with OA [21], underpinned by fear of lifelong pain, and of becoming disabled. Victor et al. [21] tested knowledge of 170 patients with OA and found that 51% agreed with the statement ‘most people with osteoarthritis end up in a wheelchair’.

General Practitioners have reported giving patients advice on likely outcomes, but in the same study avoidance of the term ‘osteoarthritis’ for fear of upsetting patients, appeared to be associated with a perception by GPs that OA does in fact have a poor outcome [23].

Some General Practitioner interview findings do concur with the patients’ reports regarding consultation experience, with some GPs holding the belief that OA is a normal part of ageing and inevitable [24]. GPs have also clearly expressed the view that OA is ‘not a disease’ [16,24] and in some instances, that there was therefore not a need for patient education [16].

General Practitioners have reported reasons for not giving written information, including lack of availability of quality resources and limited time [31]. Time in the consultation has been reported as a barrier to information giving in other UK studies [16,31], but did not appear to be an issue in a non-UK European study [32]. GPs have also reported their own knowledge needs as a barrier to information provision [24,28,33].

### Management of condition

In considering management, a number of studies referred to priorities, barriers, and challenges in treating patients with OA.

For patients, pain management and fear of disability have been reported as consultation priorities [32]. Jinks et al. [20] reported that patients tended to make their own decisions about medications, implying that consultations did not seem to contain lengthy discussions about the pros and cons of medication. Gignac et al. [17] report patient concerns that medication masks rather than cures symptoms and dissatisfaction with the amount of explanation accompanying prescriptions. Fear of side effects is reported [24,32] and the presence of co-morbidities has also been described as contributing to patient hesitancy to take medication, in addition, again to suboptimal communication around prescriptions [34]. Throughout these studies is a recurring belief among patients that they receive inadequate information and communication around prescriptions, and Alami et al. describe this as leading to suspicion of drugs [24]. Alami et al. also describe patient expectations, with those with more chronic symptoms seeking ‘cure’. Patients describe physicians communicating treatment options as ‘palliative’, causing patients to question the efficacy of ‘modern medicine’ [24].

Two studies of patient experience suggest practitioner focus on pharmacological intervention is ‘unwelcome’, suggesting patients want more information about other approaches [30,34].

Patients in focus groups discussed the inconsistency in advice regarding referral for joint replacement [16]. Patients also expressed having inadequate knowledge to make choices about surgery and anxiety about feeling the decision was theirs [16]. Patients have reported care for OA to be reactive, and not proactive with some expressing difficulty in knowing when to return to the doctor for follow-up [16].

GPs feel that patient led follow up is appropriate [23], particularly if they also hold the view that OA is ‘not a disease’ [35]. Interestingly, this belief seemed to underpin a reluctance to refer to self-management programmes, with GPs not identifying OA a chronic disease with the same standing as diabetes, but as a condition with little or no opportunity for modification of outcomes [36].

General Practitioners also report pain control as the biggest challenge in a survey of OA management in the UK [31]. GPs in this study identified practice and logistical barriers to managing pain such as lack of specialist teams and time in the consultation, in addition to lack of training. In a German study, GPs talked about specific patient barriers to managing pain; for example, they reported patients either did not accept paracetamol as a treatment due to its common use or had already tried it [32]. Rosemann et al. [32] also described a reluctance among GPs to prescribe opiates for OA, considering that patients would automatically reject these ‘heavy’ drugs, in addition to GPs perceiving opiates were ‘over-treatment’ for OA.

With regard to lifestyle change such as promotion of exercise and weight loss, GPs have described getting patients to change their lifestyle as challenging [31] and described patients as generally unwilling to change, having ‘learned to live’ with their symptoms [32]. GPs have also expressed uncertainty regarding exercise prescriptions [28]. Lambert et al. [28] highlights the different perspectives of patients and physicians; in their study doctors were reported as valuing surgical options and medication in OA treatment, with the implication non-pharmacological, non-surgical treatments were less valued by physicians, than patients.

### OA and the doctor-patient relationship

The need for doctors to value or legitimise symptoms emerges strongly from published studies [19,22], with patients in one study describing that they have not been taken seriously [24]. Patients report feeling OA is not a priority [30].

Patients described the importance of the doctor-patient relationship in the study by Alami et al. [24] and the need for doctors to be patient centred. Kee [37] describes participants with OA ‘stay[ing] in charge’ by not taking medications recommended by GPs, or not seeing doctors again who had recommended joint replacement, when this was not favoured by the patient. However, this also represents a breakdown in communication and shared decision-making. As previously mentioned, Davis et al. [34] found that patients reported communication and unmet expectations as barriers to effective pain management, in addition to personal barriers such as comorbidities and emotional distress.

General Practitioners have reported feeling that the lack of therapeutic options or cure in OA threatens the doctor-patient relationship [18,23]. Further evidence of this comes from GP reports of either requesting X-Rays or referring patients to secondary care, when they don’t believe it clinically indicated, in order to preserve the relationship [23,32]. GPs may have resultant feelings of frustration [23] and feel that patients have ‘unrealistic expectations’ [28]. An alternative viewpoint is provided by Gignac et al. who suggest doctor and patient have different orientations; doctors may approach OA from a perspective of acceptance whereas patients may believe they have more power to exert control and influence over their symptoms [17]. Busby et al. [18] describe the GP as translator of knowledge, and suggest how tensions in the doctor-patient relationship may exist between biological and sociological knowledge; if a doctor has uncertainty about biological explanations he or she may favour sociological descriptors e.g. wear and tear.

GPs’ prioritisation of OA is described by Coar, with GPs reporting it as less important than other ‘life-threatening’ conditions such as ischaemic heart disease [23]. Coar [23] also discusses the notion that a common condition may be considered less important by GPs: ‘familiarity breeds contempt’.

## Conclusions

A broad range of literature has been reviewed in order to understand what happens when patients consult with osteoarthritis. A strength of this review is the breadth of included literature, including a MPhil thesis, which has been particularly useful in illuminating the GP perspective.

From the literature reviewed, a number of issues have emerged. Firstly, patient studies indicate a range of patient-perceived negative talk that may occur in the consultation. This includes the phrase ‘wear and tear’ which may have negative connotations, reporting OA is something to be lived with and nothing can be done. The negative perception of ‘wear and tear’ is likely an unintended outcome of a term that GPs may choose with the best of intentions, to avoid causing alarm. However, patient preferences for diagnostic labelling are not clear. This review also highlights that negative comments about OA may relate to the GP’s underlying beliefs that OA is ‘not a disease’ and that it is likely to deteriorate. Importantly, negative talk may not always originate from the GP with evidence that patients may hold similar views. A need for primary care to endorse a more positive view of OA has previously been identified [38] and this review serves as a useful reminder for clinical practice of the impact of negative talk in the consultation.

Secondly, this review highlights marked divergence over management, between patient and doctor. Patients may have complex expectations and fears regarding treatment that are inadequately explored in the consultation. While patients seem keen to explore non-pharmacological options, GPs report frustration and lack of knowledge around issues to do with lifestyle change. When asked about challenges to management, GPs tend to report resource issues or time in the consultation, or patient factors, whereas patients report lack of communication. Both GPs and patients have identified knowledge deficit, and it is possible that enhanced management of OA requires an approach that addresses knowledge, communication and shared decision making, which in turn may promote greater self-management [39].

Finally, this review highlights the importance to patients of feeling that their joint pain is being taken seriously and validated. GPs that hold the belief that OA is a normal change may not adequately legitimise their patients’ symptoms and engage with management approaches. The failure to adequately validate a patient’s symptoms may lead to a downward spiral of discordance within the consultation, and this finding has resonance with work with patients’ with medically unexplained symptoms [40].

In considering the limitations of this review, it is worthy of note that the majority of cited studies concentrate on deficits in quality of care, and this may reflect publication bias to some extent. Some of the studies described are over 10 years old and may not accurately reflect the issues relevant at the current time, especially in light of new insights with regard disease pathophysiology, treatment and outcomes. Furthermore, the attitudes and beliefs of patients and doctors who agree to take time to participate in research about OA may not be representative of the population as a whole. Some of the qualitative research included had only brief mentions of a consultation with a GP, and it is possible that some of the views elicited were not entirely based on consultation experiences.

Patient preferences around the labelling of the condition, the nature of doctor explanations of osteoarthritis and discussion around management options (including the degree of shared decision making) have emerged as areas for further research. Given the limitations of the studies reviewed, observational research would be well placed to explore these issues further. Observing the consultation, and matching patient and doctor behaviours and reactions will go much further in unlocking the important ‘chain of events’, and the origin of any negative talk. In the meantime, practicing GPs may wish to reflect on the influence of negative statements on patient outcome, and whether training needs exist around the management of patients with osteoarthritis. Communication within the consultation is clearly linked to patient satisfaction, but whether interventions to enhance the doctor patient interaction influence other health outcomes remains to be seen [41].

## Abbreviations

GP: General Practitioner; NHS: National Health Service (United Kingdom); OA: Osteoarthritis.

## Competing interests

The authors declare that they have no competing interests.

## Authors’ contributions

ZP conceived the review, conducted literature searches, conducted the narrative review and drafted the manuscript. TS and ABH contributed to the narrative review and the manuscript. All authors read and approved the final manuscript.

## Author information

ZP is a Clinical Lecturer and Honorary Consultant Rheumatologist and conducted this review as part of a PhD using mixed methods to explore the osteoarthritis consultation in primary care. This is part of a larger programme of work aiming to enhance the care of osteoarthritis in primary care. TS is a Senior Research fellow with an interest in consultation research, and supervisor to ZP. ABH is a Professor of Medical Education and Consultant Rheumatologist and PhD supervisor to ZP.

## Pre-publication history

The pre-publication history for this paper can be accessed here:

http://www.biomedcentral.com/1471-2296/15/46/prepub
